# Quantifying susceptibility of marine invertebrate biocomposites to dissolution in reduced pH

**DOI:** 10.1098/rsos.190252

**Published:** 2019-06-05

**Authors:** Matthew Chadwick, Elizabeth M. Harper, Anaëlle Lemasson, John I. Spicer, Lloyd S. Peck

**Affiliations:** 1 Department of Earth Sciences, University of Cambridge, Downing Street, Cambridge CB2 3EQ, UK; 2 School of Biological and Marine Sciences, University of Plymouth, Drake Circus, Plymouth PL4 8AA, UK; 3 British Antarctic Survey, High Cross, Madingley Road, Cambridge CB3 0ET, UK

**Keywords:** ocean acidification, microstructure, bivalves, dissolution, crustaceans

## Abstract

Ocean acidification threatens many ecologically and economically important marine calcifiers. The increase in shell dissolution under the resulting reduced pH is an important and increasingly recognized threat. The biocomposites that make up calcified hardparts have a range of taxon-specific compositions and microstructures, and it is evident that these may influence susceptibilities to dissolution. Here, we show how dissolution (thickness loss), under both ambient and predicted end-century pH (approx. 7.6), varies between seven different bivalve molluscs and one crustacean biocomposite and investigate how this relates to details of their microstructure and composition. Over 100 days, the dissolution of all microstructures was greater under the lower pH in the end-century conditions. Dissolution of lobster cuticle was greater than that of any bivalve microstructure, despite its calcite mineralogy, showing the importance of other microstructural characteristics besides carbonate polymorph. Organic content had the strongest positive correlation with dissolution when all microstructures were considered, and together with Mg/Ca ratio, explained 80–90% of the variance in dissolution. Organic content, Mg/Ca ratio, crystal density and mineralogy were all required to explain the maximum variance in dissolution within only bivalve microstructures, but still only explained 50–60% of the variation in dissolution.

## 1. Introduction

The rising levels of atmospheric and oceanic *p*CO_2_ causing ocean acidification (OA) are predicted to lower seawater pH from approximately 8.1 to approximately 7.6 by 2100 [1]. The lower pH will reduce the saturation state of calcium carbonate in the oceans, presenting a direct threat to marine calcifiers by increasing the risk of dissolution, as well as inhibiting the production, of their mineralized components [2–5]. Due to the ecological and economic importance of many marine calcifiers (e.g. commercial shellfishing), there has been extensive work examining the potential effects of OA on shell production across a range of taxa [6–9]. The results of these studies differ, probably due to the complexity and number of variables in biological systems. By contrast, fewer studies have considered the impact of shell dissolution on maintaining shell integrity, especially important in longer-lived taxa [10,11]. While skeletal costs are a relatively small part of a calcifier's energy budget, when compared with metabolic costs, end-century pH conditions have been shown to substantially increase the skeletal costs in multiple bivalve species [12]. This study focuses on the susceptibility of different calcified skeletons to dissolution under acidified conditions, independent of any mediation that might occur in a living organism, and thus to explore which shell types are most vulnerable.

Skeletal hardparts are hierarchical biocomposites consisting of a stiff mineralized component embedded in a softer, organic matrix [13,14]. The detailed microstructural arrangements of these biocomposites are very varied with differences presumably driven by selection pressures (mechanical strength, density, ease of deposition and resistance to dissolution/corrosion) [15] with different taxa prioritizing different qualities [16]. Here, we address susceptibility to dissolution in acidified seawaters, although such chemical attack may also result from activities of drilling predators [17] or endolithic organisms [18] that bore into the shell. Factors affecting shell dissolution—measured as thickness loss in this study—include the relative solubility of both the mineralized and organic phases. While inorganic calcite is 35% less soluble than aragonite [19,20], other factors, such as crystal size [15], crystallographic orientation [21], Mg/Ca ratio [22] and the presence of organic material both within and between crystals [15,23,24], may also be important predictors of dissolution. A high proportion of organic matrix might either protect the mineral component or itself be preferentially lost, releasing acid residues [24] or simply causing shell disintegration through crystal loss rather than dissolution [25].

Molluscan shell microstructures have long been of interest because of their extraordinary diversification over the last 540 million years and also the ecological and economic importance of many mollusc species. Bivalve microstructures are the most diverse within this phylum, all shells being made of one or more microstructures arranged in discrete layers, with either calcite or aragonite mineralized components. The crystals show a wide range of morphologies and sizes and are arranged in dramatically different amounts of organic matrix (0.1–12 wt%) [26,27]. Crustaceans, another economically and ecologically important group, have a chitin-based cuticle strengthened by calcite and amorphous calcium carbonate (ACC) [28]. The chitin composition of crustacean cuticle makes it an interesting comparison with the dominantly mineralogical composition of bivalve shells. We investigate dissolution of a range of common bivalve microstructures and compare them with that of a crustacean using both ambient and predicted end-century seawater pHs.

Specifically, we test the following hypotheses:

— The predicted pH for end-century seawater will lead to greater dissolution of all microstructures.
— Mineralogy is the most important determinant of shell loss, with calcitic microstructures being more resistant.
— Other factors, such as low organic contents, higher Mg/Ca ratios and smaller crystals with the associated high surface area : volume, also promote dissolution.
— Shell loss is observable as etching on the mineral component as opposed to attacking the organic matrix.

## 2. Methodology

### 2.1. Material

We selected bivalve taxa that provided seven key microstructures widespread among the class (nacre, composite prisms, ‘homogeneous’ and crossed-lamellar (all aragonitic), calcitic prisms (columnar and fibrous) and calcitic foliae) [26] (table 1). We also tested the calcitic carapace of the European lobster. Material was obtained (table 1) from living organisms and kept wet throughout preparation in order to ensure that the natural relationship between the mineral and matrix components was maintained. Representative sections of each microstructure were characterized (organic content, crystal density and Mg/Ca).

**Table 1.** Source, microstructure and mineralogy for the eight sampled species.

mineralogy and microstructure	taxon used	source of materials
aragonite: nacre	internal layer of *Anodonta cygnea* (swan mussel) [29]	Maidenhead Aquatics, Cambridge, UK
aragonite: composite prisms	external layer of *Ruditapes philippinarum* (Manilla clam) [30]	Cambridge Market, UK
aragonite: crossed-lamellar	external layer of *Ensis ensis* (razor clam) [31]	Portland Shellfisheries, UK
aragonite: ‘homogeneous’	external layer of *Arctica islandica* [32]	Millport, Scotland, UK
calcite: columnar prisms	external layer of *Atrina pectinata* [33]	Cape d'Aguilar, Hong Kong
calcite: fibrous prisms	external layer of *Mytilus edulis* (blue mussel) [34]	Cambridge Market, UK
calcite: foliae	external layer of *Pecten maximus* (king scallop) [35]	Portland Shellfisheries, UK
chitin microfibres reinforced with calcite and ACC	claw of *Homarus gammarus* (European lobster) [28]	Portland Shellfisheries, UK

### 2.2. Microstructure characterization

Organic content was determined using thermogravimetric analysis (TGA), where weight loss on accurate temperature ramping (100–500°C) allows material proportions to be identified. We ran 5–15 mg ground samples (from which all adherent periostracum had been removed) on a TAQ500 following the protocol of Harper *et al*. [36]. Crystal morphology and distribution of organic material were determined by scanning electron microscopy (SEM) of fractured or polished and etched samples (using a QEMSCAN 650F at accelerating voltages of 10 kV) following a similar method to Bieler *et al*. [26]. Micrographs of three random 50 µm^2^ areas from polished samples were used to calculate the number of exposed crystals/area (crystal density). Crystal units were defined for the composite prisms by the larger prismatic units rather than the individual fibres [26] as they define the dominant crystal edges in the microstructure. Similarly, for the crossed-lamellar, we measured the first-order lamellae as the major units (e.g. [37]). For the crystal density of lobster, we measured the density of pores in the chitin lattice as an equivalent metric. The relative concentrations of magnesium and calcium were determined on polished samples by an electron nanoprobe analysis on a Cameca SX100.

### 2.3. Dissolution experiment

For each microstructure, 30 samples were embedded in individual polyester resin blocks, before being ground and polished down to a 9 µm grit to expose shell (free of any periostracum) with the desired microstructure at the surface. All of the polished blocks were kept moist to ensure the organics did not dry out. Fifteen blocks per microstructure were exposed to one of two pH treatments: ambient (pH 7.89) and reduced (pH 7.65). The OA mesocosm system used during the experiment was set up in the University of Plymouth and was the one described in Lemasson *et al*. [38] (environmental parameter variations in electronic supplementary material, A). Briefly, each of the two treatments consisted of an 80 l seawater header tank aerated with either ambient air (*p*CO_2_ ≈ 400 ppm; pH ≈ 7.89) or CO_2_-enriched air (*p*CO_2_ ≈ 1000 ppm; pH ≈ 7.65). CO_2_ levels in the gas supplies were recorded using a CO_2_ analyser (LI-820; LI-COR, Lincoln, NE, USA) and adjusted manually to the desired level twice daily. Seawater was gravity-fed from each of the header tanks into six 3 l replicate tanks at a constant rate of approximately 60 ml min^−1^. The 15 blocks for each microstructure and treatment were divided between the six replicate tanks. Excess seawater was allowed to overflow from the tanks to the sump, where it was filtered, aerated and recirculated to the header tanks. Seawater in the system originated from Plymouth Sound (UK) and, following mechanical filtering and UV sterilization, was added and replaced on a daily basis to account for evaporation. Filtration and UV treatment resulted in only partial sterilization, enough to allow the build-up, but not over-accumulation, of microbial life. Deionized water was added as needed to maintain stable salinity levels. Temperature, salinity and pH were measured daily in all replicate tanks as described in Lemasson *et al*. [38]. Total alkalinity was measured once a week in each of the replicate tanks (details in electronic supplementary material, A).

All blocks were removed after 100 days exposure. One block from each microstructural type and treatment was retained to study patterns of dissolution by SEM (as above). The remainder were used to estimate the amount of dissolution. This was achieved in a similar way to that described in Kennish & Lutz [39] whereby the block was topped up by further resin, sawn along central lines and thickness loss relative to the initial resin surface was measured using the graticule of an optical microscope. A minimum of 30 measurements were taken for each microstructure and treatment.

### 2.4. Statistics

The dissolution between treatments was compared and related to the properties for each microstructure. Two-way ANOVAs were used to compare dissolution between treatments for each microstructure (electronic supplementary material, B, table S1). Multiple regression analysis (electronic supplementary material, B, table S2) was used to investigate the relative importance of each microstructural characteristic in relation to shell dissolution. All of the statistical tests were conducted using Microsoft Excel.

## 3. Results

**Figure 1.**
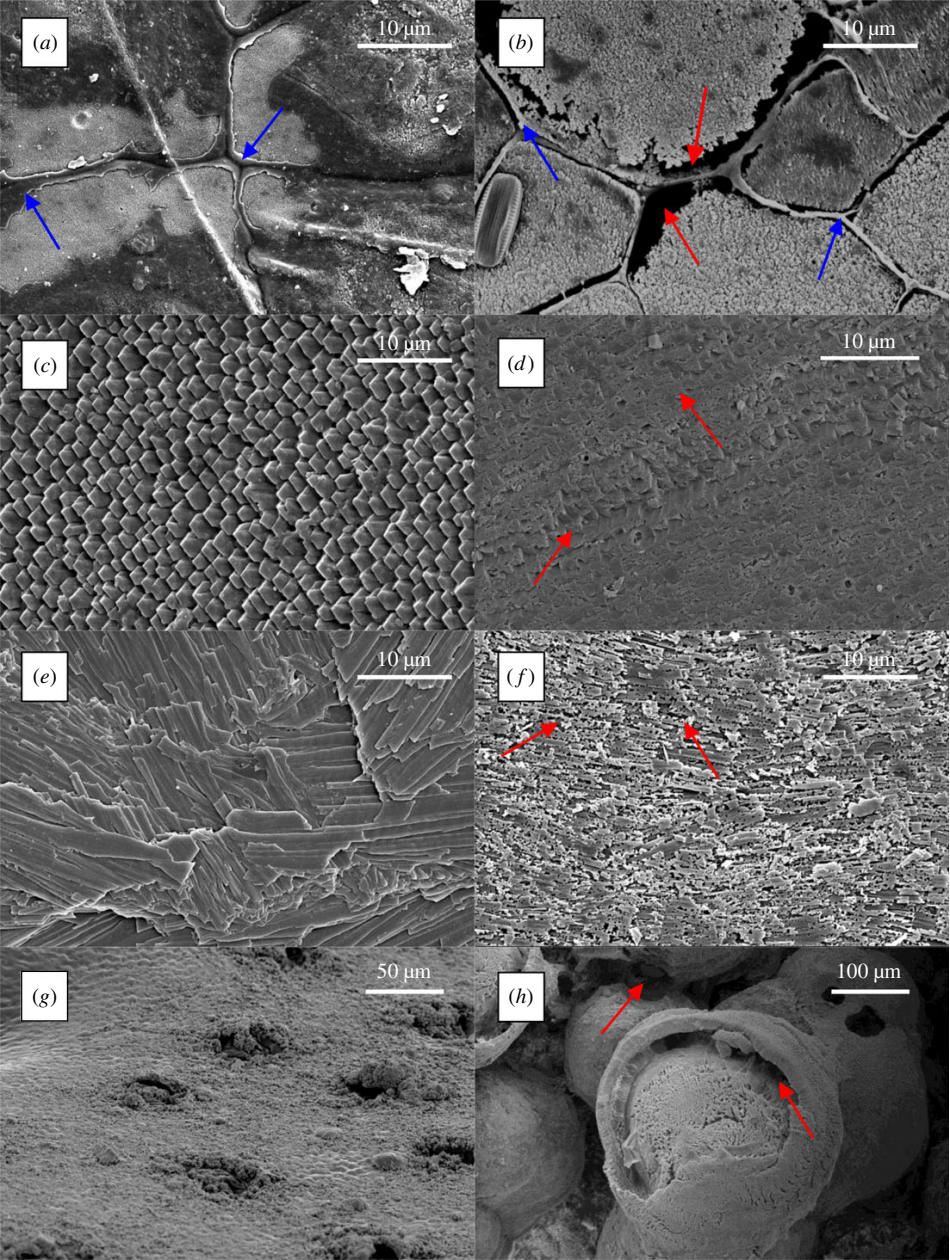
SEM images of the calcite microstructures—columnar prisms (*a,b*), fibrous prisms (*c,d*), foliae (*e,f*) and chitin microfibres reinforced with calcite and ACC (*g,h*)—before (left—*a,c,e,g*) and after (right—*b,d,f,h*) 100 days under reduced pH. Red arrows indicate areas of dissolution after 100 days. Blue arrows indicate the organic jackets around the columnar prisms (*a*,*b*).

**Figure 2.**
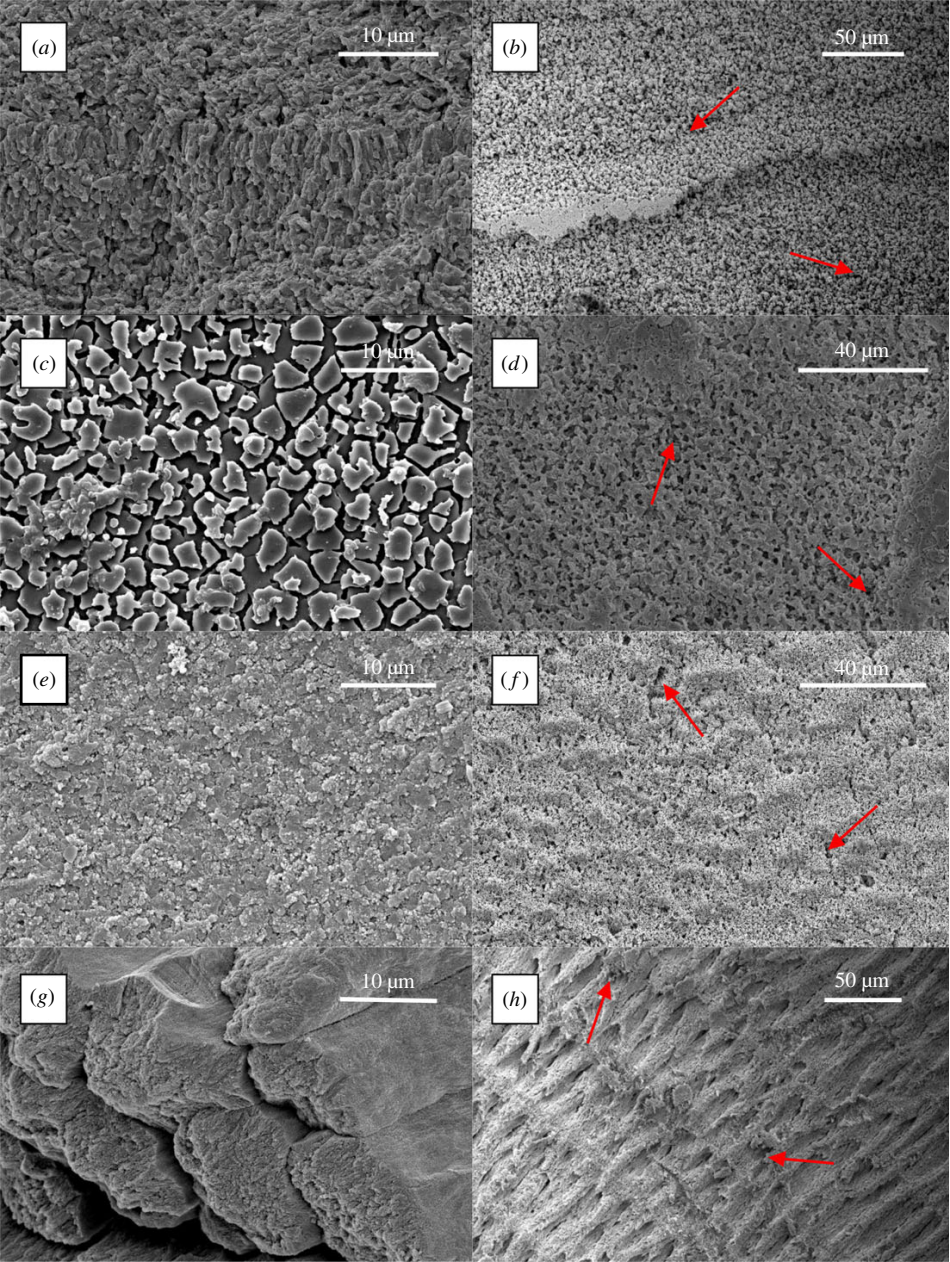
SEM images of the aragonite microstructures—‘homogeneous’ (*a,b*), nacre (*c,d*), crossed-lamellar (*e,f*) and composite prisms (*g,h*)—before (left—*a,c,e,g*) and after (right—*b,d,f,h*) 100 days under reduced pH. Red arrows indicate areas of dissolution after 100 days.

**Figure 3.**
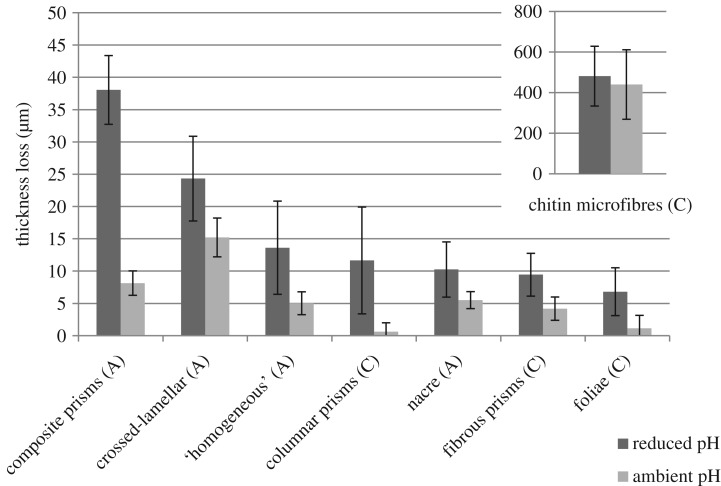
Thickness loss for ambient and reduced pH treatments. The mineralogy is given in brackets after each microstructure. Error bars are ±1 s.d. Note the different ordinate axis scales for the main plot and the inset for the European lobster.

All of the sampled microstructures showed clear signs of dissolution under ambient and reduced pH conditions (visual observations; figures 1 and 2). Under both treatments, there was an order of magnitude greater dissolution of the lobster cuticle, which lost most of the total thickness (visual observations), than any of the bivalve microstructures (figure 3). The dissolution for all the bivalves was greater at the lower pH (*p* ≪ 0.01). The ordering of dissolution susceptibility was largely the same between treatments, although composite aragonite prisms and crossed-lamellar aragonite swapped positions as the most susceptible bivalve microstructure (figure 3) and columnar calcite prisms were much more resistant to dissolution under the ambient pH conditions (figure 3).

SEM (figures 1 and 2) revealed dissolution of the originally polished surfaces. Our observation of the pattern of dissolution (marked by red arrows in figures 1 and 2) shows it occurs preferentially along grain boundaries between crystals. In all the samples at reduced pH, the organic envelopes have been removed at the same rate as the neighbouring mineralized components. This is clearest in figure 1*a,b* where the organic envelope (marked by the blue arrows) of the *Atrina pectinata* is level with the surface of the prisms both before and after dissolution. By contrast, the organic jackets are more resistant than the prisms in *A. pectinata* when etched with dilute HCl (M. Chadwick 2017, personal observation).

Organic content (table 2) strongly and positively correlated with dissolution under both treatments (*p* ≪ 0.01) when all eight species were included in the regression (electronic supplementary material, B, table S2). There was also a significant correlation (*p* < 0.01) if the lobster and nacre were excluded. There was no significant correlation for Mg/Ca ratio (table 2) in the bivalves (*p* = 0.33), but there was a strong positive correlation with dissolution when lobster cuticle was included in the regression (*p* ≪ 0.01). There was a negative trend for crystal density (table 2) within the bivalves, but the relationship was not significant (*p* = 0.18).

**Table 2.** Microstructural characteristics. Values given as mean ± 1 s.d.

microstructure	measured crystal density (crystals/(50 µm)^2^) (*n* = 3)	measured organic content (wt%) (*n* = 4)	measured Mg/Ca ratio^a^ (*n* = 3)
nacre (aragonite)	155 ± 7	4.2 ± 0.6	0
composite prisms (aragonite)	5.7 ± 1.5	2.32 ± 0.08	0
crossed-lamellar (aragonite)	100 ± 10	2.17 ± 0.06	0
‘homogeneous’ (aragonite)	790 ± 210	1.79 ± 0.06	0
columnar prisms (calcite)	3.0 ± 0.5	1.5 ± 0.1	0.0101 ± 0.0006
fibrous prisms (calcite)	850 ± 190	1.42 ± 0.06	0.0021 ± 0.0003
foliae (calcite)	1700 ± 200	1.1 ± 0.1	0.0055 [40]
lobster cuticle (calcite and ACC with α-chitin)	240 ± 10	22 ± 2	0.051 ± 0.003

^a^Only the calcitic microstructures have [Mg] above the detection limit for the nanoprobe (approx. 100 ppm). Foliae is the only calcitic sample with a zero [Mg] and for the analysis, an Mg/Ca value of 0.0055 is taken from Freitas *et al*. [40].

Multiple regression analysis values (electronic supplementary material, B, table S2) show that when all eight microstructures were included, organic content and Mg/Ca were the best predictors of dissolution (*p* ≪ 0.01) under both treatments, and when combined, they explained 80–90% of the variance in dissolution. For both treatments, organic content of the skeleton was the more important of the two predictors. When considering just the bivalve microstructures, only 50–60% of the variance in dissolution could be explained by the measured factors and for the reduced pH treatment, the most significant correlation (*p* ≪ 0.01) required the inclusion of organic content, Mg/Ca, crystal density and mineralogy as predictors. For the ambient pH treatment, only organic content, Mg/Ca and crystal density were required to get the most significant correlation (*p* ≪ 0.01), notably not mineralogy. For reduced pH, the most important predictor was mineralogy, whereas for ambient pH, it was the Mg/Ca ratio.

## 4. Discussion

The susceptibility of molluscs to shell loss in undersaturated waters is well known in fresh water [41], cold waters [23], deep sea [39] and natural CO_2_ vents [42], hence the concern for these, and other calcifying organisms, under predicted end-century pH conditions. We sought to investigate whether certain biocomposites were more resistant to such loss and therefore which species will be required to invest more energy in shell maintenance under OA.

Even during a mollusc's lifetime, damage to the periostracum, the outer protective membrane, is common [43]. By removing the periostracum from our samples (or the epicuticle on the lobster samples), we have investigated the susceptibility of the underlying microstructure to dissolution under acidified conditions. Under future OA, there is a chance that calcifying organisms will be able to alleviate this dissolution through targeted compensatory skeleton growth. However, additional skeletal growth has an associated energy cost and thus species with more susceptible skeletal microstructures are likely to have a reduced fitness under OA.

As hypothesized, our experiments showed that even over a 100-day period, dissolution was evident as thickness loss and corroded surfaces in both ambient and reduced pH treatments, and were greater in the latter. Of particular interest is whether, based on their inorganic properties [19,20], calcitic shells are more resistant to dissolution than aragonitic ones. Calcitic layers have evolved multiple times within both the bivalve and gastropod molluscs, particularly in epifaunal shallow water taxa where such layers have always been added to the outside of the shell. This has been suggested as an adaptation against shell dissolution in undersaturated habitats [23,44,45]. However, in our experiment, although calcite was found to be the most important predictor of dissolution-resistance under reduced pH among bivalves, it only explained 27% of the variance in the data (electronic supplementary material, B). Most surprising was the observation that the calcitic lobster cuticle was the most susceptible microstructure. This may be explained by the high levels of metastable ACC in the endocuticle [28], where the skeleton consists of three parts, the epicuticle, the exocuticle and the endocuticle. These high dissolution rates of lobster cuticle could also explain why decapod crustaceans have a relatively poor fossil record [46].

The quantity and distribution of the organic component is important because greater amounts of organics might be expected to shield the mineralized components from contact with the seawater and inhibit dissolution. Alternatively, organics might actually enhance dissolution by promoting the growth of microbes that release acids [24] or facilitate the disaggregation of mineral grains by the loss of binding organics [25,47]. The distribution of organics should also have an effect because more unevenly distributed organics will result in more localized enhancement/inhibition of the dissolution. In our study, the multiple regression analysis emphasized the importance of organic content as a predictor for dissolution. The high dissolution of the lobster cuticle may be related to its ACC content rather than its organic content. However, the bivalve microstructures, with nacre excluded, also show a significant positive correlation (*p* ≪ 0.01) between organic content and dissolution. Within the bivalve microstructures tested, the organic content of nacre was twice that of the others, but the more homogeneous distribution of organics, with each nacre tablet having its own protein envelope [48], probably increased resistance and reduced the relative levels of dissolution. The discrepancy between the seeming susceptibility of organics in the mesocosm and their resilience under etching could be explained by the presence of microbes in seawater, supported by the prolific evidence for endolithic algae boring in the more organic-rich nacre (E.M. Harper 2017, personal observation).

The mesocosm seawater is not fully ‘natural’ due to the sterilization and filtering in the experimental set-up. However, the partial sterilization is necessary to keep overproliferation of microbial life from occurring over exposure periods of weeks and months. The presence of biofilms and endolithic algae boring on the post-dissolution samples (M. Chadwick & E.M. Harper 2017, personal observation) indicates that the conditions were not completely sterile. In unsterilized seawater, with a higher microbe activity, high organic contents would be expected to further promote dissolution than in sterile conditions. Therefore, while the conditions within the mesocosm are an important consideration, our conclusions are still expected to be a faithful, if possibly conservative, representation of ‘natural’ seawater conditions.

The Mg/Ca ratio would be expected to influence dissolution because calcite with a higher Mg content has been shown to be less stable in aqueous solutions [22]. This is supported in this study by the strong positive correlation with dissolution when all the samples were considered. However, this relationship was probably due to the strong statistical pull caused by lobster cuticle having much higher values for both dissolution and Mg/Ca than any of the bivalves. When the analysis was restricted to only the bivalves, the Mg/Ca ratio had no significant influence on shell dissolution, although it did account for the most variance (approx. 42%) under ambient conditions.

Crystal size and orientation might be expected to have an effect on solubility because dissolution should preferentially occur along crystal edges, and so microstructures with larger crystals or with crystals orientated to show a larger ‘face’ at the surface will have a lower density of grain boundaries and should be more resistant to dissolution. However, although the SEM evidence showed preferential loss of material along crystal boundaries, the relationship with dissolution was not significant (*p* = 0.18), in contrast with previous work [15]. On the other hand, low crystal densities were generally related to higher organic contents and so the effect from the organics may have overridden any crystal density effect.

## 5. Conclusion

All sampled species suffered significantly higher dissolution under lower pH, but the lobster skeleton was much more susceptible to dissolution than any of the bivalves. Within the bivalves, calcite mineralogies were generally more resistant to dissolution, but organic content, Mg/Ca and crystal density were also important factors affecting susceptibility. By contrast, dissolution variation between all microstructures was most strongly correlated with just organic content and Mg/Ca ratio, with high organic content as the main predictor for higher dissolution. This counters the commonly held view that organic matter within the shell should act to shield the mineralogy from dissolution, with the possible exception of nacre, where data suggested the distribution of organics is important and not just the absolute amount (wt%). Although the majority of the variance in dissolution across all microstructures could be explained by the predictors studied here, within just the bivalves, there was almost 50% variation that remains unexplained. Factors that could be involved include crystallographic orientation of the mineralogy and the composition of the organic phase. Notwithstanding the effects of having living organisms within the shells, the seemingly greater susceptibility of lobster to OA could have important ecological consequences for predator–prey interactions in the future.

## Supplementary Material

Mesocosm Environmental Data and TA Methodology

Reviewer comments

## Supplementary Material

Statistical Analysis

## Data accessibility

Data (values for microstructural characteristics (mineralogy, crystal density, organic content, Mg/Ca ratio) and thickness loss under both pH treatments (ambient and reduced)) are available from the Dryad Digital Repository at: https://doi.org/10.5061/dryad.mm05699 [49].
