# Supplementary material for: Quantifying susceptibility of marine invertebrate biocomposites to dissolution in reduced pH
Source: R Soc Open Sci. 2019 Jun 5;6(6):190252. doi: 10.1098/rsos.190252 (PMC6599774; doi:10.1098/rsos.190252)
Supplement: Mesocosm Environmental Data and TA Methodology [file rsos190252supp1.docx]

*Supplementary A – Mesocosm Environmental Data*

**Supplementary Figure 1:** Variations in mesocosm environmental parameters (pH, temperature, salinity, total alkalinity and *p*CO_2_) over a period of 100 days.

Total alkalinity measurement methodology:

Total alkalinity was calculated by transferring 125 mL water samples to a borosilicate bottle with Teflon caps and poisoned with 30 μL of saturated HgCl_2_ solution (0.02 % sample volume) before being kept in the dark until measurement by automatic Gran titration (Titralab AT1000 © Hach Company).
