# Supplementary material for: Quantifying susceptibility of marine invertebrate biocomposites to dissolution in reduced pH
Source: R Soc Open Sci. 2019 Jun 5;6(6):190252. doi: 10.1098/rsos.190252 (PMC6599774; doi:10.1098/rsos.190252)
Supplement: Statistical Analysis [file rsos190252supp2.docx]

*Supplementary B – Statistical Analysis*

**Supplementary Table 1:** Two-way ANOVA values comparing thickness loss measurements between the two pH treatments for each microstructure. P values that show significant difference between the treatments (at the 95% confidence interval) are in bold.

|  | ANOVA values |
| --- | --- |
| Nacre (Aragonite) | F = 34.23  **p = 1.61e-7** |
| Composite prisms (Aragonite) | F = 1084.31  **p = 1.51e-45** |
| Crossed-lamellar (Aragonite) | F = 62.93  **p = 1.18e-11** |
| ‘Homogeneous’ (Aragonite) | F = 40.40  **p = 2.92e-8** |
| Columnar prisms (Calcite) | F = 72.52  **p = 7.21e-13** |
| Fibrous prisms (Calcite) | F = 71.03  **p = 2.21e-12** |
| Foliae (Calcite) | F = 46.62  **p = 6.15e-9** |
| Lobster cuticle (Calcite and α-chitin) | F = 1.30  p = 0.26 |

|  | Ambient  All Samples | Ambient  Bivalves Only | Reduced  All Samples | Reduced  Bivalves Only |
| --- | --- | --- | --- | --- |
| Organic Content | R^2^=0.839  Sig. F = 5.3e-112 | R^2^=0.087  Sig. F = 3.2e-6 | R^2^=0.881  Sig. F = 7.1e-142 | R^2^=0.016  Sig. F = 0.041 |
| Mg/Ca | R^2^=0.805  Sig. F = 2.1e-100 | R^2^=0.415  Sig. F = 1.6e-29 | R^2^=0.846  Sig. F = 1.7e-124 | R^2^=0.144  Sig. F = 1.8e-10 |
| Crystal Density | R^2^=0.017  Sig. F = 0.031 | R^2^=0.130  Sig. F = 9.0e-9 | R^2^=0.030  Sig. F = 0.002 | R^2^=0.236  Sig. F = 4.6e-17 |
| Mineralogy | R^2^=0.122  Sig. F = 1.9e-9 | R^2^=0.439  Sig. F = 1.0e-31 | R^2^=0.107  Sig. F = 5.6e-9 | R^2^=0.268  Sig. F = 1.4e-19 |
| Organic Content + Mg/Ca | R^2^=0.844  Sig. F = 4.3e-112 | R^2^=0.417  Sig. F = 1.8e-28 | R^2^=0.888  Sig. F = 1.1e-143 | R^2^=0.151  Sig. F = 4.8e-10 |
| Organic Content + Crystal Density | R^2^=0.841  Sig. F = 6.4e-111 | R^2^=0.150  Sig. F = 4.4e-9 | R^2^=0.882  Sig. F = 1.3e-140 | R^2^=0.257  Sig. F = 1.4e-17 |
| Organic Content + Mineralogy | R^2^=0.843  Sig. F = 1.6e-111 | R^2^=0.477  Sig. F = 4.3e-34 | R^2^=0.884  Sig. F = 9.1e-142 | R^2^=0.361  Sig. F = 3.4e-26 |
| Mg/Ca + Crystal Density | R^2^=0.806  Sig. F = 6.4e-99 | R^2^=0.531  Sig. F = 1.2e-39 | R^2^=0.849  Sig. F = 2.4e-124 | R^2^=0.343  Sig. F = 1.2e-24 |
| Mg/Ca + Mineralogy | R^2^=0.832  Sig. F = 9.8e-108 | R^2^=0.475  Sig. F = 7.0e-34 | R^2^=0.880  Sig. F = 2.4e-139 | R^2^=0.272  Sig. F = 9.1e-19 |
| Crystal Density + Mineralogy | R^2^=0.191  Sig. F = 1.9e-13 | R^2^=0.447  Sig. F = 3.6e-31 | R^2^=0.212  Sig. F = 2.7e-16 | R^2^=0.340  Sig. F = 2.4e-24 |
| Organic Content + Mg/Ca + Crystal Density | R^2^=0.845  Sig. F = 7.1e-111 | R^2^=0.602  Sig. F = 5.7e-47 | R^2^=0.888  Sig. F = 3.9e-142 | R^2^=0.497  Sig. F = 1.1e-38 |
| Organic Content + Mg/Ca + Mineralogy | R^2^=0.844  Sig. F = 1.3e-110 | R^2^=0.510  Sig. F = 2.4e-36 | R^2^=0.889  Sig. F = 1.4e-142 | R^2^=0.366  Sig. F = 1.2e-25 |
| Organic Content + Crystal Density + Mineralogy | R^2^=0.843  Sig. F = 3.7e-110 | R^2^=0.499  Sig. F = 3.5e-35 | R^2^=0.884  Sig. F = 3.4e-140 | R^2^=0.500  Sig. F = 5.0e-39 |
| Mg/Ca + Crystal Density + Mineralogy | R^2^=0.837  Sig. F = 8.5e-108 | R^2^=0.531  Sig. F = 1.3e-38 | R^2^=0.882  Sig. F = 5.8e-139 | R^2^=0.349  Sig. F = 3.3e-24 |
| Organic Content + Mg/Ca + Crystal Density + Mineralogy | R^2^=0.845  Sig. F = 1.2e-109 | R^2^=0.609  Sig. F = 1.0e-46 | R^2^=0.889  Sig. F = 1.8e-141 | R^2^=0.525  Sig. F = 5.9e-41 |

**Supplementary Table 2:** Multiple regression analysis values for the two pH treatments both with and without the inclusion of lobster cuticle. Every combination of the measured microstructural characteristics is considered with the strongest predictor combination highlighted in yellow.
