# Supplementary material for: Quantifying susceptibility of marine invertebrate biocomposites to dissolution in reduced pH
Source: R Soc Open Sci. 2019 Jun 5;6(6):190252. doi: 10.1098/rsos.190252 (PMC6599774; doi:10.1098/rsos.190252)
Supplement: Reviewer comments [file rsos190252_review_history.pdf]

THE ROYAL SOCIETY
PUBLISHING

ROYAL SOCIETY
OPEN SCIENCE

Quantifying susceptibility of marine invertebrate biocomposites to dissolution in reduced pH

Matthew Chadwick, Elizabeth M. Harper, Anaëlle Lemasson, John I. Spicer and Lloyd S. Peck

Article citation details
R. Soc. open sci. **6**: 190252.
http://dx.doi.org/10.1098/rsos.190252

Review timeline

Original submission:	11 February 2019
Revised submission:	5 May 2019
Final acceptance:	13 May 2019

Note: Reports are unedited and appear as submitted by the referee. The review history appears in chronological order.

Note: This manuscript was transferred from another Royal Society journal without peer review.

Review History

RSOS-190252.R0 (Original submission)

Review form: Reviewer 1

Is the manuscript scientifically sound in its present form?
Yes

Are the interpretations and conclusions justified by the results?
Yes

Is the language acceptable?
Yes

Is it clear how to access all supporting data?
Yes

Do you have any ethical concerns with this paper?
No

Reports © 2019 The Reviewers; Decision Letters © 2019 The Reviewers and Editors; Responses © 2019 The Reviewers, Editors and Authors. Published by the Royal Society under the terms of the Creative Commons Attribution License http://creativecommons.org/licenses/by/4.0/, which permits unrestricted use, provided the original author and source are credited

Have you any concerns about statistical analyses in this paper?
No

Recommendation?
Accept with minor revision (please list in comments)

Comments to the Author(s)
This interesting study addresses the susceptibility of several bivalves and one lobster microstructures under normal and acidified conditions.
It is well written. The experimental setting and the statistical analyses are competent.
The conclusions are interesting and worth being published.
Nevertheless, I have a few concerns with the study, particularly with regards to the introduction of the lobster cuticle in the study, which biases all conclusions when the whole set of samples is considered. It shows by far the largest amount of organic matter and this leads to the false idea that organic content may influence the rate of dissolution, when it is clear that the critical factor is the extremely labile ACC content. Although this is discussed in the text, this point should be stressed further.
<u>Detailed comments</u>
Line 44.- A reference is needed for this last statement.
Lines 127.- Fig. 1D is not such good. Should be improved.
Lines 136-137.- Dissolution of organic envelopes at the same rate as the crystals is not evident from Fig. 1A, B.
Lines 150-151.- This statement is not evident in Table 2. Refer to Suppl. Material Table 2.
Table 2.- I have concerns with some of the values here. Crystal density in, at least crossed lamellar and composite aragonitic prisms, depends very much on what you consider a crystallite unit is. From the data provided, I presume that authors have taken into account the size of the prismatic units in composite prisms and the sizes of 1st order lamellae in crossed lamellar, which the actual crystallite size may be much smaller: individual fibers in the former and 3rd order lamellae in the latter. This has to be taken into account. Another issue that strikes me is the low organic content in *Atrina* (1.5%). How many analyses have the authors performed to estimate the organic content? I tend to think that the value is highly underestimated for this microstructure. Finally, lobster cuticle (calcite and α-chitin) should be changed to calcite and ACC plus α-chitin or alike, to give an account of how different is this microstructure from the rest.
Lines 213-216.- These conclusions might have to be changed if, as I expect, the amount of organic matter in Atrina changed to higher values.

Review form: Reviewer 2

Is the manuscript scientifically sound in its present form?
Yes

Are the interpretations and conclusions justified by the results?
Yes

Is the language acceptable?
Yes

Is it clear how to access all supporting data?
Yes

Do you have any ethical concerns with this paper?
No

Have you any concerns about statistical analyses in this paper?
No

Recommendation?
Accept with minor revision (please list in comments)

Comments to the Author(s)
The susceptibility of the shells of calcifying marine organisms to mass loss during life is an important issue given levels of ocean acidification expected by the end of the century, and controlled experiments such as described here will be an important component of understanding that phenomenon. This ms is a scholarly treatment of the issues and of past work, and has a good scope and structure, including the reactions of 7 bivalve species – each typifying a different major shell microstructural type across aragonite and calcite common to mollusks – and arthropod cuticle, which is less heavily calcified but typical of crustacean shellfish. Both current and 'reduced pH' conditions are considered, using standard methods of measuring shell mass loss (change in thickness) after exposure to natural seawater charged with pCO2 to acidify it to target levels. I was concerned that the methods included sterilizing that SW (with UV), significantly altering conditions from natural ones by eliminating a major factor in shell disintegration via microbial maceration of the shell's organic matrix. Both Harper (2000) and Glover & Kidwell (1993), both cited here, tested explicitly the effects of sterilizing seawater on shell persistence, and found significantly lower loss rates (and different patterns of microstructural deterioration) than when using fully natural, 'microbially active' SW. I think the narrow, sterile conditions of these new experiments has in fact undercut the broader value of the results, where very little of the variation in observed loss rates could be explained by the variables considered. I must say also that none of the authors' 'after' photos resembled SEM-resolution damage that I've observed on shells drawn from modern seabeds, even high-latitude seabeds subject to low-saturated overlying water – the experimentally produced damage here only resembles lab-induced dissolution under sterile conditions. A comment within the discussion acknowledges this discrepancy, referring to pers obs of intense endolithic algae in nacre from natural seawater (the refs above could also be cited). The experimentally generated damage is thus worrisome, including the observation that organic matrix retreated to the same degree as the mineral phase in their experimental blocks – that is as hard to explain as is the low power of shell attributes to explain variation in shell loss. Overall -- Despite extensive attention to calcifiers under OA conditions, little work has actually been done on microstructural consequences for the carbonate skeleton itself, and so the paper remains a valuable and publishable contribution despite my concerns RE set up – it is internally consistent, well documented, and robust. It should, however, be strengthened by clarifying the limitations of the experimental conditions – a paragraph in the discussion is needed. Some reasoning for the choice to sterilize the mesocosm would be helpful to other investigators – was it to reduce the variables, or necessary to protect equipment?

Decision letter (RSOS-190252.R0)

29-Apr-2019

Dear Mr Chadwick

On behalf of the Editors, I am pleased to inform you that your Manuscript RSOS-190252 entitled "Quantifying Susceptibility of Marine Invertebrate Biocomposites to Dissolution in Reduced pH"

has been accepted for publication in Royal Society Open Science subject to minor revision in accordance with the referee suggestions. Please find the referees' comments at the end of this email.

The reviewers and handling editors have recommended publication, but also suggest some minor revisions to your manuscript. Therefore, I invite you to respond to the comments and revise your manuscript.

• Ethics statement
If your study uses humans or animals please include details of the ethical approval received, including the name of the committee that granted approval. For human studies please also detail whether informed consent was obtained. For field studies on animals please include details of all permissions, licences and/or approvals granted to carry out the fieldwork.

• Data accessibility
It is a condition of publication that all supporting data are made available either as supplementary information or preferably in a suitable permanent repository. The data accessibility section should state where the article's supporting data can be accessed. This section should also include details, where possible of where to access other relevant research materials such as statistical tools, protocols, software etc can be accessed. If the data has been deposited in an external repository this section should list the database, accession number and link to the DOI for all data from the article that has been made publicly available. Data sets that have been deposited in an external repository and have a DOI should also be appropriately cited in the manuscript and included in the reference list.

If you wish to submit your supporting data or code to Dryad (http://datadryad.org/), or modify your current submission to dryad, please use the following link:
http://datadryad.org/submit?journalID=RSOS&manu=RSOS-190252

• Competing interests
Please declare any financial or non-financial competing interests, or state that you have no competing interests.
• Authors' contributions
All submissions, other than those with a single author, must include an Authors' Contributions section which individually lists the specific contribution of each author. The list of Authors should meet all of the following criteria; 1) substantial contributions to conception and design, or acquisition of data, or analysis and interpretation of data; 2) drafting the article or revising it critically for important intellectual content; and 3) final approval of the version to be published.

All contributors who do not meet all of these criteria should be included in the acknowledgements.

We suggest the following format:
AB carried out the molecular lab work, participated in data analysis, carried out sequence alignments, participated in the design of the study and drafted the manuscript; CD carried out the statistical analyses; EF collected field data; GH conceived of the study, designed the study, coordinated the study and helped draft the manuscript. All authors gave final approval for publication.

• Acknowledgements
Please acknowledge anyone who contributed to the study but did not meet the authorship criteria.

• Funding statement
Please list the source of funding for each author.

Please ensure you have prepared your revision in accordance with the guidance at https://royalsociety.org/journals/authors/author-guidelines/ -- please note that we cannot publish your manuscript without the end statements. We have included a screenshot example of the end statements for reference. If you feel that a given heading is not relevant to your paper, please nevertheless include the heading and explicitly state that it is not relevant to your work.

Because the schedule for publication is very tight, it is a condition of publication that you submit the revised version of your manuscript before 08-May-2019. Please note that the revision deadline will expire at 00.00am on this date. If you do not think you will be able to meet this date please let me know immediately.

To revise your manuscript, log into https://mc.manuscriptcentral.com/rsos and enter your Author Centre, where you will find your manuscript title listed under "Manuscripts with Decisions". Under "Actions," click on "Create a Revision." You will be unable to make your revisions on the originally submitted version of the manuscript. Instead, revise your manuscript and upload a new version through your Author Centre.

When submitting your revised manuscript, you will be able to respond to the comments made by the referees and upload a file "Response to Referees" in "Section 6 - File Upload". You can use this to document any changes you make to the original manuscript. In order to expedite the processing of the revised manuscript, please be as specific as possible in your response to the referees. We strongly recommend uploading two versions of your revised manuscript:

1) Identifying all the changes that have been made (for instance, in coloured highlight, in bold text, or tracked changes);
2) A 'clean' version of the new manuscript that incorporates the changes made, but does not highlight them.

When uploading your revised files please make sure that you have:

1) A text file of the manuscript (tex, txt, rtf, docx or doc), references, tables (including captions) and figure captions. Do not upload a PDF as your "Main Document";
2) A separate electronic file of each figure (EPS or print-quality PDF preferred (either format should be produced directly from original creation package), or original software format);
3) Included a 100 word media summary of your paper when requested at submission. Please ensure you have entered correct contact details (email, institution and telephone) in your user account;
4) Included the raw data to support the claims made in your paper. You can either include your data as electronic supplementary material or upload to a repository and include the relevant doi within your manuscript. Make sure it is clear in your data accessibility statement how the data can be accessed;
5) All supplementary materials accompanying an accepted article will be treated as in their final form. Note that the Royal Society will neither edit nor typeset supplementary material and it will be hosted as provided. Please ensure that the supplementary material includes the paper details where possible (authors, article title, journal name).

Supplementary files will be published alongside the paper on the journal website and posted on the online figshare repository (https://rs.figshare.com/). The heading and legend provided for each supplementary file during the submission process will be used to create the figshare page,

so please ensure these are accurate and informative so that your files can be found in searches. Files on figshare will be made available approximately one week before the accompanying article so that the supplementary material can be attributed a unique DOI.

Please note that Royal Society Open Science charge article processing charges for all new submissions that are accepted for publication. Charges will also apply to papers transferred to Royal Society Open Science from other Royal Society Publishing journals, as well as papers submitted as part of our collaboration with the Royal Society of Chemistry (http://rsos.royalsocietypublishing.org/chemistry).

If your manuscript is newly submitted and subsequently accepted for publication, you will be asked to pay the article processing charge, unless you request a waiver and this is approved by Royal Society Publishing. You can find out more about the charges at http://rsos.royalsocietypublishing.org/page/charges. Should you have any queries, please contact openscience@royalsociety.org.

Once again, thank you for submitting your manuscript to Royal Society Open Science and I look forward to receiving your revision. If you have any questions at all, please do not hesitate to get in touch.

Kind regards,
Andrew Dunn
Royal Society Open Science Editorial Office
Royal Society Open Science
openscience@royalsociety.org

on behalf of Prof Kevin Padian (Subject Editor)
openscience@royalsociety.org

Associate Editor Comments to Author:

Two reviewers have provided substantial feedback on your paper, but recommend publication once you have addressed these concerns. Please ensure that you not only respond in the manuscript to their commentary but provide a point-by-point response when you submit the revision.

Reviewer comments to Author:
Reviewer: 1

Comments to the Author(s)
This interesting study addresses the susceptibility of several bivalves and one lobster microstructures under normal and acidified conditions.
It is well written. The experimental setting and the statistical analyses are competent.
The conclusions are interesting and worth being published.
Nevertheless, I have a few concerns with the study, particularly with regards to the introduction of the lobster cuticle in the study, which biases all conclusions when the whole set of samples is considered. It shows by far the largest amount of organic matter and this leads to the false idea that organic content may influence the rate of dissolution, when it is clear that the critical factor is the extremely labile ACC content. Although this is discussed in the text, this point should be stressed further.
<u>Detailed comments</u>

Line 44.- A reference is needed for this last statement.
Lines 127.- Fig. 1D is not such good. Should be improved.
Lines 136-137.- Dissolution of organic envelopes at the same rate as the crystals is not evident from Fig. 1A, B.
Lines 150-151.- This statement is not evident in Table 2. Refer to Suppl. Material Table 2.
Table 2.- I have concerns with some of the values here. Crystal density in, at least crossed lamellar and composite aragonitic prisms, depends very much on what you consider a crystallite unit is. From the data provided, I presume that authors have taken into account the size of the prismatic units in composite prisms and the sizes of 1st order lamellae in crossed lamellar, which the actual crystallite size may be much smaller: individual fibers in the former and 3rd order lamellae in the latter. This has to be taken into account. Another issue that strikes me is the low organic content in *Atrina* (1.5%). How many analyses have the authors performed to estimate the organic content? I tend to think that the value is highly underestimated for this microstructure. Finally, lobster cuticle (calcite and α-chitin) should be changed to calcite and ACC plus α-chitin or alike, to give an account of how different is this microstructure from the rest.
Lines 213-216.- These conclusions might have to be changed if, as I expect, the amount of organic matter in Atrina changed to higher values.

Reviewer: 2

Comments to the Author(s)
The susceptibility of the shells of calcifying marine organisms to mass loss during life is an important issue given levels of ocean acidification expected by the end of the century, and controlled experiments such as described here will be an important component of understanding that phenomenon. This ms is a scholarly treatment of the issues and of past work, and has a good scope and structure, including the reactions of 7 bivalve species – each typifying a different major shell microstructural type across aragonite and calcite common to mollusks – and arthropod cuticle, which is less heavily calcified but typical of crustacean shellfish. Both current and 'reduced pH' conditions are considered, using standard methods of measuring shell mass loss (change in thickness) after exposure to natural seawater charged with pCO2 to acidify it to target levels. I was concerned that the methods included sterilizing that SW (with UV), significantly altering conditions from natural ones by eliminating a major factor in shell disintegration via microbial maceration of the shell's organic matrix. Both Harper (2000) and Glover & Kidwell (1993), both cited here, tested explicitly the effects of sterilizing seawater on shell persistence, and found significantly lower loss rates (and different patterns of microstructural deterioration) than when using fully natural, 'microbially active' SW. I think the narrow, sterile conditions of these new experiments has in fact undercut the broader value of the results, where very little of the variation in observed loss rates could be explained by the variables considered. I must say also that none of the authors' 'after' photos resembled SEM-resolution damage that I've observed on shells drawn from modern seabeds, even high-latitude seabeds subject to low-saturated overlying water – the experimentally produced damage here only resembles lab-induced dissolution under sterile conditions. A comment within the discussion acknowledges this discrepancy, referring to pers obs of intense endolithic algae in nacre from natural seawater (the refs above could also be cited). The experimentally generated damage is thus worrisome, including the observation that organic matrix retreated to the same degree as the mineral phase in their experimental blocks – that is as hard to explain as is the low power of shell attributes to explain variation in shell loss. Overall -- Despite extensive attention to calcifiers under OA conditions, little work has actually been done on microstructural consequences for the carbonate skeleton itself, and so the paper remains a valuable and publishable contribution despite my concerns RE set up – it is internally consistent, well documented, and robust. It should, however, be strengthened by clarifying the limitations of the experimental conditions – a paragraph in the discussion is needed. Some

reasoning for the choice to sterilize the mesocosm would be helpful to other investigators – was it to reduce the variables, or necessary to protect equipment?

Author's Response to Decision Letter for (RSOS-190252.R0)

See Appendix A.

Decision letter (RSOS-190252.R1)

13-May-2019

Dear Mr Chadwick,

I am pleased to inform you that your manuscript entitled "Quantifying Susceptibility of Marine Invertebrate Biocomposites to Dissolution in Reduced pH" is now accepted for publication in Royal Society Open Science.

You can expect to receive a proof of your article in the near future. Please contact the editorial office (openscience_proofs@royalsociety.org and openscience@royalsociety.org) to let us know if you are likely to be away from e-mail contact. Due to rapid publication and an extremely tight schedule, if comments are not received, your paper may experience a delay in publication.

Royal Society Open Science operates under a continuous publication model (http://bit.ly/cpFAQ). Your article will be published straight into the next open issue and this will be the final version of the paper. As such, it can be cited immediately by other researchers. As the issue version of your paper will be the only version to be published I would advise you to check your proofs thoroughly as changes cannot be made once the paper is published.

On behalf of the Editors of Royal Society Open Science, we look forward to your continued contributions to the Journal.

Kind regards,
Royal Society Open Science Editorial Office
Royal Society Open Science
openscience@royalsociety.org

on behalf of Kevin Padian (Subject Editor)
openscience@royalsociety.org

Follow Royal Society Publishing on Twitter: @RSocPublishing
Follow Royal Society Publishing on Facebook:
https://www.facebook.com/RoyalSocietyPublishing.FanPage/
Read Royal Society Publishing's blog: https://blogs.royalsociety.org/publishing/

Appendix A

Response to reviewers' comments

We are very grateful for the constructive and kind reviews provided by the two anonymous reviewers of our manuscript. The comments are encouraging and we have sought to respond to them with revisions and clarifications. Please see below, in blue, our detailed response to the comments.

Reviewer 1:

This interesting study addresses the susceptibility of several bivalves and one lobster microstructures under normal and acidified conditions. It is well written. The experimental setting and the statistical analyses are competent. The conclusions are interesting and worth being published.

Nevertheless, I have a few concerns with the study, particularly with regards to the introduction of the lobster cuticle in the study, which biases all conclusions when the whole set of samples is considered. It shows by far the largest amount of organic matter and this leads to the false idea that organic content may influence the rate of dissolution, when it is clear that the critical factor is the extremely labile ACC content. Although this is discussed in the text, this point should be stressed further.

We have added clarification (lines 212-214) to reiterate the importance of ACC rather than organic content in the lobster cuticle. We have also stressed that the bivalves show the same trend between organic content and dissolution.

"The high dissolution of the lobster cuticle may be related to its ACC content rather than its organic content. However, the bivalve microstructures, with nacre excluded, also show a significant positive correlation ($p<<0.01$) between organic content and dissolution."

Detailed comments

Line 44.- A reference is needed for this last statement.
Taylor & Layman 1972 has now been added.

Lines 127.- Fig. 1D is not such good. Should be improved.
The post dissolution SEM photo has been replaced with one that more clearly shows the dissolution of the prisms.

Lines 136-137.- Dissolution of organic envelopes at the same rate as the crystals is not evident from Fig. 1A, B.
We have added a sentence of clarification (lines 142-144) to the main text which explicitly refers to what the reader should see, and take away from, Figures 1A and 1B.

Lines 150-151.- This statement is not evident in Table 2. Refer to Suppl. Material Table 2.
This reference has been updated.

Table 2.- I have concerns with some of the values here. Crystal density in, at least crossed lamellar and composite aragonitic prisms, depends very much on what you consider a crystallite unit is. From the data provided, I presume that authors have taken into account the size of the prismatic units in composite prisms and the sizes of 1st order lamellae in crossed lamellar, which the actual crystallite size may be much smaller: individual fibers in the former and 3rd order lamellae in the latter. This has to be taken into account.

For assessing the risk from OA we suggest that the complete composite prisms and the 1st order lamellae are the fundamental blocks that will experience dissolution along their perimeters. However, we appreciate that this decision requires clarification and have added an explanation for these two microstructures, with accompanying references, to the methodology (lines 91-94).

Another issue that strikes me is the low organic content in *Atrina* (1.5%). How many analyses have the authors performed to estimate the organic content? I tend to think that the value is highly underestimated for this microstructure.

For the organic content of each microstructure 4 analyses were performed using Thermogravimetric Analysis. The *Atrina* returned values of 1.46, 1.59, 1.35, 1.51 which are all consistently around 1.5%. This value is also not substantially less than values in the published literature (e.g. Harper 2000). Consequently, we have revised Table 2 so that the number of analyses for each microstructural characteristic is made clear.

Finally, lobster cuticle (calcite and α-chitin) should be changed to calcite and ACC plus α-chitin or alike, to give an account of how different is this microstructure from the rest.
This has been corrected.

Lines 213-216.- These conclusions might have to be changed if, as I expect, the amount of organic matter in Atrina changed to higher values.
If we changed the *Atrina* organic content to the slightly higher values cited in the literature (≈1.8%) it doesn't change the overall conclusions or the outlier nature of the nacre organic content.

Reviewer 2:

The susceptibility of the shells of calcifying marine organisms to mass loss during life is an important issue given levels of ocean acidification expected by the end of the century, and controlled experiments such as described here will be an important component of understanding that phenomenon. This ms is a scholarly treatment of the issues and of past work, and has a good scope and structure, including the reactions of 7 bivalve species – each typifying a different major shell microstructural type across aragonite and calcite common to mollusks – and arthropod cuticle, which is less heavily calcified but typical of crustacean shellfish. Both current and 'reduced pH' conditions are considered, using standard methods of measuring shell mass loss (change in thickness) after exposure to natural seawater charged with pCO2 to acidify it to target levels. I was concerned that the methods included sterilizing that SW (with UV), significantly altering conditions from natural ones by eliminating a major factor in shell disintegration via microbial maceration of the shell's organic matrix. Both Harper (2000) and Glover & Kidwell (1993), both cited here, tested explicitly the effects of sterilizing seawater on shell persistence, and found significantly lower loss rates (and different patterns of microstructural deterioration) than when using fully natural, 'microbially active' SW. I think the narrow, sterile conditions of these new experiments has in fact undercut the broader value of the results, where very little of the variation in observed loss rates could be explained by the variables considered. I must say also that none of the authors' 'after' photos resembled SEM-resolution damage that I've observed on shells drawn from modern seabeds, even high-latitude seabeds subject to low-saturated overlying water – the experimentally produced damage here only resembles lab-induced dissolution under sterile conditions. A comment within the discussion acknowledges this discrepancy, referring to pers obs of intense endolithic algae in nacre from natural seawater (the refs above could also be cited). The experimentally generated damage is thus worrisome, including the observation that organic matrix retreated to the same degree as the mineral phase in their experimental blocks – that is as hard to explain as is the low power of shell attributes to explain variation in shell loss.

We thank the reviewer for their kind comments on the manuscript. We acknowledge the importance of making clear the limitations of our experimental setup and have sought to clarify this in lines 221-228. The 'sterilisation' technique employed results only in a partial sterilisation of the water, but enough to make sure that the material in the exposure mesocosm is not 'overwhelmed' by bacterial growth over an exposure period of weeks and months. There was substantial evidence from the samples, and from background measurements made when the mesocosm was used for testing live invertebrates (J.I. Spicer, unpublished observations) that the mesocosm was not actually fully sterile. We have therefore argued that whilst the non-natural conditions are an important caveat (although not sterilizing the water also results in non-natural conditions, i.e. dominance of microbes in the system) they don't substantially affect our conclusions.

"The mesocosm seawater is not fully 'natural' due to the sterilisation and filtering in the experimental setup. However, the partial sterilisation is necessary to keep overproliferation of microbial life from occurring over exposure periods of weeks and months. The presence of biofilms and endolithic algae boring on the post-dissolution samples (personal observation) indicates that the conditions were not completely sterile. In unsterilized seawater, with a higher microbe activity, high organic contents would be expected to further promote dissolution than in sterile conditions. Therefore, whilst the conditions within the mesocosm are an important consideration, our conclusions are still expected to be a faithful, if possibly conservative, representation of 'natural' seawater conditions."

Overall -- Despite extensive attention to calcifiers under OA conditions, little work has actually been done on microstructural consequences for the carbonate skeleton itself, and so the paper remains a valuable and publishable contribution despite my concerns RE set up – it is internally consistent, well documented, and robust. It should, however, be strengthened by clarifying the limitations of the experimental conditions – a paragraph in the discussion is needed. Some reasoning for the choice to sterilize the mesocosm would be helpful to other investigators – was it to reduce the variables, or necessary to protect equipment?

We have added a sentence to the methodology (lines 113-114) to explain that the partial sterilisation and filtering are required to prevent the overproliferation of microbial life which has been observed to occur in the mesocosm otherwise. The same experimental setup is used to investigate the effect of OA and warming on living organisms, and without the partial sterilisation, there is significant accumulation of microbial life, and a greater prevalence of disease in the test subjects.
